# Species identification of juvenile fishes of the genus *Pseudoblennius* using mitochondrial DNA barcoding

**DOI:** 10.1080/23802359.2018.1456982

**Published:** 2018-03-28

**Authors:** Hyuck Joon Kwun

**Affiliations:** National Marine Biodiversity Institute of Korea, Seocheon, Korea

**Keywords:** Barcoding, *Pseudoblennius*, identification, Korea, juvenile

## Abstract

Species identification is important in natural science and should be precise. Six specimens of juvenile *Pseudoblennius* were collected from the eastern coastal waters of the Korean Peninsula and Jeju Island in 2016–2017, and identified for the first time using DNA barcoding based on mitochondrial DNA cytochrome oxidase subunit I sequences. DNA barcoding analysis supported three adult species of genus *Pseudoblennius* (*P. cottoides*, *P. marmoratus*, and *P. percoides*) being quite distinct from each other. Six juvenile specimens were completely identified: two as *P. cottoides*; two more as *P. marmoratus*; and the final two as *P. percoides*. Mitochondrial DNA COI can be effective as a means of species identification method for the genus *Pseudoblennius*.

Accurate species identification in natural science is important because misidentification has a serious negative effect on scientific results or decisions (Austen et al. 2016), such as mistaken identification of endangered species (Hunt 2015) and errors in species monitoring (Culverhouse et al. 2003).

Identification of marine fish larvae or juveniles is a prerequisite for understanding a species’ life history, for which morphological characters have traditionally been used (Blaxter 1984). However, a problem with morphology-based identification is that many fishes exhibit the same or duplicated characters (Victor et al. 2009; Ko et al. 2013). In particular, closely related taxa, such as congeneric species and cryptic species, can be difficult to identify on the basis of morphology (Taylor and Watson 2004; Matarese et al. 2011). Thus, various new methods have emerged to solve this problem, including DNA barcoding.

DNA barcoding is a fast and easy method of species identification for taxonomic experts or non-experts using a single gene sequence (Hebert et al. 2003). The greatest benefit of DNA barcoding is that it can fill lacunae in morphological identification, given an understanding of ecology and evolution (Ko et al. 2013; Kress et al. 2015; Bhattacharya et al. 2016). Therefore, DNA barcoding is widely used in the identification of larval and juvenile marine fishes (Victor 2007; Paine et al. 2008; Hubert et al. 2010; Ji et al. 2017).

The sculpins family Cottidae is represented by about 70 genera and 282 species on the coasts of the Pacific Ocean (Nelson et al. 2016). They exhibit great diversity in both morphology and ecology, and most species occur in the intertidal region to the continental slope in the North Pacific (Hastings et al. 2014). The genus *Pseudoblennius* (Temminck and Schlegel 1850) contains only six nominal species distributed throughout the coastal waters of Korea and Japan in the northwestern Pacific (Nakabo and Kai 2013; Echmeyer et al. 2017). Of these six species, four have been reported from Korea (Kim et al. 2005): *Pseudoblennius cottoides* (Richardson 1848), *Pseudoblennius marmoratus* (Steindachner and Döderlein 1884), *Pseudoblennius percoides* (Günther 1861), and *Pseudoblennius zonostigma* (Jordan and Starks 1904). Although previous studies have provided morphological descriptions of larvae and juveniles (Yoo et al. 2003; Okiyama 2014), species in this genus are difficult to identify because early growth stages show similar shape, colouration pattern, and meristic characters. Also, indoor-reared early stage specimens exhibit a different morphology from natural specimens of the same species (Leis and Carson-Ewart 2000). Therefore, biochemical methods are necessary for accurate species identification, particularly for wild-captured specimens. The aim of the present study was to identify wild-captured juvenile specimens of the genus *Pseudoblennius* using DNA barcoding of the mitochondrial DNA cytochrome oxidase subunit I (COI) region, and to provide barcode information for this genus for the first time.

Six juvenile specimens of the genus *Pseudoblennius* were collected from the eastern coastal waters of the Korean Peninsula and Jeju Island in 2016–2017 (Figure 1; Table 1). For comparison, three adult *Pseudoblennius* species (*P. cottoides*, *P. marmoratus*, and *P. percoides*) were collected from the eastern coast of Jeju Island, Korea (Table 1); identification of these taxa was based on morphology, following Nakabo and Kai (2013). *Furcina osimae* (family Cottidae) was selected as an outgroup species. All specimens were fixed as whole-body specimens in 99% ethanol, and have been deposited at the Marine Fish Diversity (MFD) of the National Marine Biodiversity Institute of Korea.

**Figure 1.**
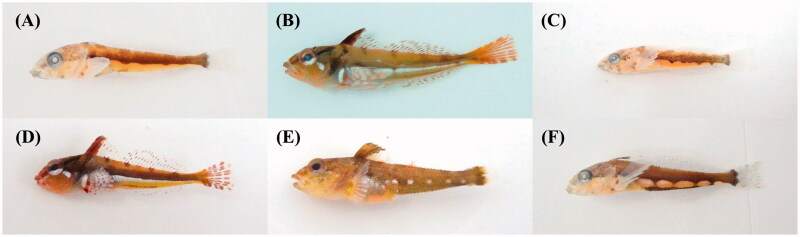
The photography of six juvenile *Pseudoblennius* specimens. (A) *Pseudoblennius* sp. 1, MFD-590; (B) *Pseudoblennius* sp. 2, MFD-673; (C) *Pseudoblennius* sp. 3, MFD-926; (D) *Pseudoblennius* sp. 4, MFD-927; (E) *Pseudoblennius* sp. 5, MFD-931; (F) *Pseudoblennius* sp. 6, MFD-949.

**Table 1.** List of specimens.

Species	Locality	Voucher no.	Accession no.
Juvenile			
* Pseudoblennius* sp. 1	Ulsan, Korea	MFD-590	MG922924
* Pseudoblennius* sp. 2	Jeju Island, Korea	MFD-673	MG922927
* Pseudoblennius* sp. 3	Jeju Island, Korea	MFD-926	MG922931
* Pseudoblennius* sp. 4	Jeju Island, Korea	MFD-927	MG922928
* Pseudoblennius* sp. 5	Jeju Island, Korea	MFD-931	MG922932
* Pseudoblennius* sp. 6	Samcheok, Korea	MFD-949	MG922926
Adult			
* Pseudoblennius cottoides*	Jeju Island, Korea	MFD-901	MG922925
* Pseudoblennius marmoratus*	Jeju Island, Korea	MFD-1024	MG922929
* Pseudoblennius percoides*	Jeju Island, Korea	MFD-672	MG922930
Outgroup			
* Furcina osimae*	Ulsan, Korea	MFD-607	MG922933

The juvenile specimens were identified by means of the DNA barcoding protocol described by Ward et al. (2005). Genomic DNA was extracted from the right-side eye using a DNeasy Blood and Tissue Kit (Qiagen, Hilden, Germany). Mitochondrial DNA COI was amplified using a universal primer set (VF2_t1 and FishR2_t1) (Ward et al. 2005). The nucleotide sequences of all specimens (juvenile, adult, and outgroup specimens) have been deposited in the DDBJ/EMBL/GenBank databases (accession numbers: MG922924–922933). The sequences were aligned with ClustalW (Thompson et al. 1994) in BioEdit ver. 7 (Hall 1999). The genetic distances were calculated and a neighbour-joining (NJ) tree was produced using MEGA 7 (Kumar et al. 2016), based on the Kimura two-parameter model (Kimura 1980) and 10,000 bootstrap replications.

Mitochondrial DNA COI sequences for six juveniles and three adults of the genus *Pseudoblennius* were obtained. Based on an analysis of 618 base pairs (bp), interspecific genetic distances (*d*) calculated for three adult species of *Pseudoblennius* were between 0.012 and 0.125. Comparing juveniles and adults, *Pseudoblennius* sp. 1 corresponds to *P. cottoides* (genetic distance *d* = 0.000), but differs from *P. percoides* (*d* = 0.077) and *P. marmoratus* (*d* = 0.125). *Pseudoblennius* sp. 2 is similar to *P. marmoratus* (*d* = 0.002), but differs from *P. percoides* (*d* = 0.117) and *P. cottoides* (*d* = 0.127). *Pseudoblennius* sp. 3 almost corresponds to *P. percoides* (*d* = 0.005), but is distinct from *P. cottoides* (*d* = 0.076) and *P. marmoratus* (*d* = 0.118). *Pseudoblennius* sp. 4 can be identified as *P. marmoratus* (*d* = 0.000), but differs from *P. percoides* (*d* = 0.119) and *P. cottoides* (*d* = 0.125). *Pseudoblennius* sp. 5 almost corresponds to *P. percoides* (*d* = 0.008), but differs from *P. cottoides* (*d* = 0.079) and *P. marmoratus* (*d* = 0.121). *Pseudoblennius* sp. 6 be assigned to *P. cottoides* (*d* = 0.000), but differs from *P. percoides* (*d* = 0.077) and *P. marmoratus* (*d* = 0.125).

The result of the NJ analysis is shown in Figure 2. In the NJ tree, the three adult species of genus *Pseudoblennius* are well divided into three major clades, which are supported by high bootstrap values. *Pseudoblennius* sp. 1 and sp. 6 cluster with *P. cottoides*, *Pseudoblennius* sp. 2 and sp. 4 cluster with *P. marmoratus*, and *Pseudoblennius* sp. 3 and sp. 5 cluster with *P. percoides*, all of which are corroborated by 100% bootstrap value.

**Figure 2.**
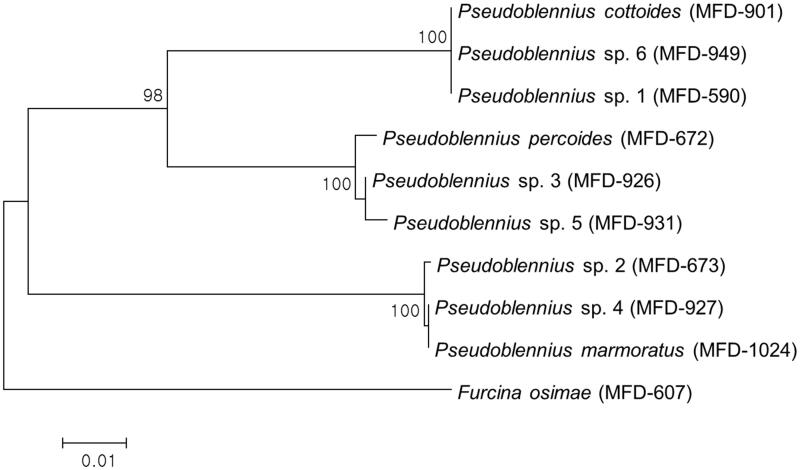
Neighbour-joining tree of mitochondrial DNA COI for three *Pseudoblennius* species including six juvenile specimens. Numbers of branches correspond to bootstrap probabilities in 10,000 bootstrap replications. Bar indicates genetic distance of 0.01.

Overall, six juvenile specimens of *Pseudoblennius* are completely identified to the species level: *Pseudoblennius* sp. 1 and sp. 6 as *P. cottoides*, *Pseudoblennius* sp. 2 and sp. 4 as *P. marmoratus*, and *Pseudoblennius* sp. 3 and sp. 5 as *P. percoides*.

The present study provides mitochondrial DNA barcoding information for species identification within the genus *Pseudoblennius* for the first time. Mitochondrial DNA COI sequence data support the species-level distinction of three *Pseudoblennius* species. Therefore, mitochondrial DNA COI can be effective as a species identification method for the genus *Pseudoblennius*. In the future, further research could focus on validating previous morphological descriptions and identifying diagnostic morphological characters for use as taxonomic keys in juvenile *Pseudoblennius* species.
